# Role of the Nervous System in the Control of Proteostasis during Innate Immune Activation: Insights from *C. elegans*

**DOI:** 10.1371/journal.ppat.1003433

**Published:** 2013-08-08

**Authors:** Alejandro Aballay

**Affiliations:** Department of Molecular Genetics and Microbiology, Duke University Medical Center, Durham, North Carolina, United States of America; The University of North Carolina at Chapel Hill, United States of America

## Introduction

Like other free-living nematodes, the one-millimeter-long nematode *Caenorhabditis elegans* lives in soils and composts rich in microorganisms, including human microbial pathogens. In the laboratory, *C. elegans* animals are typically propagated by feeding them *Escherichia coli*. This bacterium is effectively disrupted by the *C. elegans* pharyngeal grinder and essentially no intact bacterial cells can be found in the intestinal lumen of healthy, young animals. Once in the gut, however, pathogenic bacteria are capable of proliferating, invading host cells, and killing *C. elegans* by infectious processes. Bacterial pathogens can also adhere to the cuticle of the nematode, causing a defensive swelling response of the epidermal cells. While *C. elegans* lacks adaptive immunity, it responds to pathogen exposure by avoiding certain potentially pathogenic bacteria and by activating an inducible innate immune system. Thus, pathogen avoidance, grinding, swelling, peristalsis, and secretion of antimicrobial substances prevent microbial colonization of *C. elegans* by bacterial pathogens. Increasing evidence highlights the role of the *C. elegans* nervous system in the control of some of these immune responses against bacterial pathogens.

## Importance of Using *C. elegans* to Study Neural-Immune Communications

Activation of the innate immune system upon pathogen recognition results in a rapid activation of microbial killing pathways that need to be highly controlled because deficiencies or excesses in the response have deleterious consequences. Indeed, the activation of the immune system appears to account for the major physiological, metabolic, and pathological responses to infections. Furthermore, while insufficient immune responses can lead to infection and cancer, excessive immune responses have been linked to conditions such as Crohn's disease, rheumatoid arthritis, atherosclerosis, diabetes, and Alzheimer's disease. Increasing evidence indicates that metazoans take advantage of the nervous system to receive inputs from infected sites and integrate them in a reflexive manner to coordinate appropriate immune responses.

The 1986 Nobel laureate Rita Levi-Montalcini commented during her Nobel lecture how the complexity of neural-immune networks, which at that time were already known to be closely interrelated, opens endless possibilities for interventions. However, given the complexity of the nervous and immune systems of most model organisms, the precise mechanisms by which the two systems influence each other remain largely understudied. *C. elegans* is emerging as a model system that may help to fill this gap and identify neural mechanisms capable of regulating immune responses.

The nervous system of most model organisms consist of billions or hundreds of billions of neurons, whose wiring at the cellular level is far from characterized. In contrast, the information about the identity, morphology, and synaptic connectivity of each of the 302 neurons in the nervous system of *C. elegans* is known. Thus, this information provides a unique opportunity to study the activity of specific neurons involved in neural-immune communications and to study the flow of signals between neurons in response to pathogen infection.

## Neurons and Neural Signals Involved in the Control of Immune Responses in *C. elegans*

**Figure 1:**
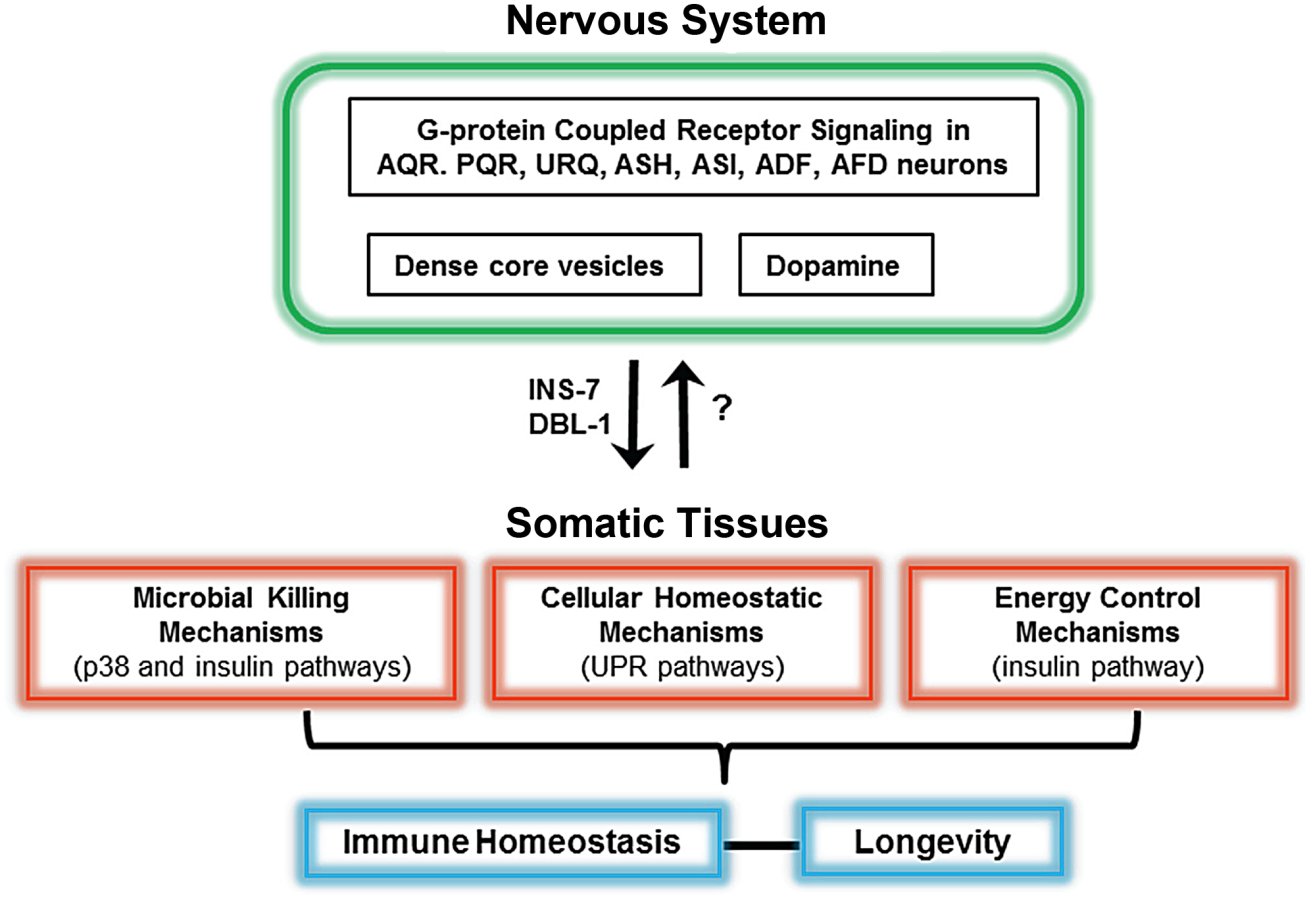
Schematic of the neural control of different pathways required for immunity in *C. *elegans. At least seven neurons in the nervous system control microbial killing pathways and cellular homeostatic pathways. In addition to AQR, PQR, URX, ASH, and ASI neurons, dopaminergic neurons also control the p38 MAPK pathway. While ASH and ASI control the unfolded protein response (UPR), ADF and AFD control both the UPR and the insulin pathway. Neurally produced INS-7 and DBL-1 target somatic tissues to control immunity. A fine-tuned control of protein homeostasis and metabolism by the nervous system seems to be important to maintain immune homeostasis.

The study of neurally expressed G-protein coupled receptors, NPR-1 and OCTR-1, highlights the role of specific neurons in the control of immune responses [1], [2]. Animals carrying mutations in the *npr-1* gene exhibit enhanced susceptibility to infections by *Pseudomonas aeruginosa*, *Salmonella enterica*, and *Enterococcus faecalis*, suggesting that NPR-1–expressing neurons control immune pathways important for defense against a broad range of pathogens. Interestingly, avoidance of certain bacterial pathogens, such as *P. aeruginosa*, is also an important defense mechanism in *C. elegans* that is regulated by the NPR-1 neural circuit [1], [3]. A full-genome expression analysis on animals with altered neural function due to mutation in *npr-1* showed an enrichment in genes that are markers of innate immune responses, most of which are expressed in the intestine and/or regulated by a conserved PMK-1/p38 mitogen-activated protein kinase (MAPK) signaling pathway [1]. This indicates that the nervous system controls not only avoidance to certain pathogens but also the expression of immune genes in somatic tissues. The use of mosaic animals expressing NPR-1 in selected cells and neurally ablated animals link NPR-1–expressing neurons AQR, PQR, and URX to the control of immunity [1] (Figure 1).

Similar studies using neuron-specific rescues and genetic ablation of neurons indicate that OCTR-1 functions in ASH and ASI neurons to suppress the p38 MAPK pathway and *unfolded protein response* (UPR) genes that are expressed in pharyngeal and intestinal cells [2], [4] (Figure 1). A unique component of GPCR signaling in *C. elegans*, the GPCR adaptor protein arrestin-1, functions downstream OCTR-1 and NPR-1 in the nervous system [5], providing further evidence for the role of AQR, PQR, URX, ASH, and ASI in the control of innate immunity. In addition, the use of mosaic animals expressing arrestin-1 only in ADF and AFD indicate that these cells also control *C. elegans* immunity [5] (Figure 1).

OCTR-1 is a catecholamine receptor related to vertebrate adrenergic receptors whose ligand is octopamine, an invertebrate equivalent of noradrenaline. In mammals, it is known that noradrenaline mediates responses to acute stress that result in a decreased immune response. The findings indicating that immunity can be activated by inhibiting a pathway involved in the arousal response suggest that there is a strong selective advantage to suppressing immune responses during stress, and that similar chemical cues in the nervous system of *C. elegans* and mammals may have similar immune consequences. In *C. elegans*, chemosensory neurons and serotonin signaling also control pathogen detection and avoidance [6], [7], but it is unclear to what extent those responses to pathogen exposure are found across metazoans. In addition to octopamine and serotonin, dopamine also appears to play a role in the control of immunity as dopaminergic neurons participate in a circuit that makes *C. elegans* more resistant to reinfections with enteropathogenic *E. coli* [8]. This dopamine-dependent resistance to reinfection mechanism requires both p38 MAPK and the insulin-like pathway that regulates lifespan and immunity [8] (Figure 1).

*C. elegans* neurons are known to express numerous secreted peptides of the transforming growth factor beta (TGF beta) family, the insulin family, and neuropeptide families. This myriad of secreted factors has the potential to act at a distance to modulate various physiological processes by regulating the function of neural and nonneural cells throughout the animal. It is known that the insulin-like neuropeptide INS-7 released from dense-core vesicles in the nervous system controls the expression of immune genes in the *C. elegans* intestinal cells [9], [10]. Moreover, the TGF beta receptor ligand DBL-1, which is produced by the nervous system, regulates the expression of immune genes that are expressed in the epidermis of *C. elegans* [11] (Figure 1).

## Role of the Nervous System in the Control of Proteostasis during Immune Activation

In metazoans, the endoplasmic reticulum (ER) is an important intracellular organelle in which newly synthesized proteins are folded, assembled, and matured. When protein homeostasis, or proteostasis, is disrupted by environmental stresses or changes in physiological conditions, a series of unfolded protein response (UPR) pathways are activated. When the increased demand for protein folding is not met with an appropriate UPR, the accumulation of damaged proteins typically results in cell death. In fully immunocompetent nematodes, bacterial infections are controlled by a range of immune effectors that are highly expressed in pharyngeal and intestinal cells. In response to bacterial infection, genes involved in the UPR are also upregulated, indicating that the increased demand for protein folding in the ER must be successfully alleviated for a complete immune response to be mounted [2], [4], [5].

To maintain organismal homeostasis in response to bacterial infections, *C. elegans* uses both canonical and noncanonical UPR pathways. The X-box binding protein 1 (XBP-1) branch of the UPR, which is the most conserved branch of the UPR across species, is activated in response to exposure to pore-forming toxins by a mechanism that requires the p38 MAPK pathway [12]. The XBP- 1 branch is also activated by exposure to *Pseudomonas aeruginosa* and it is essential for larval development in infected animals [13], [14]. A noncanonical UPR pathway that is independent of XBP-1 is also activated by *P. aeruginosa* infection, and is required for survival when the animals are infected with *P. aeruginosa* or *S. enterica* [2], [15]. This noncanonical UPR pathway comprises a family of genes encoding prion-like glutamine[Q]/asparagine[N]-rich domain-bearing proteins that are activated in *xbp-1* mutant animals when ER stress is induced by tunicamycin treatment. Functional studies demonstrate that these genes encode UPR proteins that function in parallel with the canonical UPR pathway [16].

Maintenance of protein homeostasis in response to the stress caused by *P. aeruginosa* infection is controlled at the cell nonautonomous level by OCTR-1. OCTR-1 signaling in ASH and ASI neurons controls the activation of noncanonical UPR genes that are upregulated in response to pathogen infection and strongly expressed in nonneuronal tissues such as those corresponding to the pharynx and the intestine, primary interfaces between host cells involved in immune responses and bacterial pathogens [2], [5]. Interestingly, OCTR-1 does not control the canonical XPB-1 branch of the UPR during development, but it does gain control of it during adulthood [4] (Figure 1). This temporal control of the canonical UPR pathway by the nervous system suggests that the high demand for protein folding that takes place during development does not need to be fine-tuned by cell nonautonomous mechanisms. In contrast, immune activation in adult animals seems to be tightly controlled by neural influences. The activation of the UPR in response to toxins and infections requires p38 MAPK. Further experimentation will be required to identify potential neural mechanisms that fine-tune this interaction during the immune response.

## Concluding Remarks

Animals have evolved sophisticated mechanisms to modify specific properties in response to changes such as those that take place during response to microbial infections. The nervous system, which can sense many types of environmental stimuli, appears to integrate inflammatory signals to maintain a balanced immune response against invading microorganisms. In response to pathogen infection, the *C. elegans* nervous system appears to play a role in the maintenance of organismal homeostasis by controlling killing mechanisms such as those regulated by the p38 MAPK pathway, cellular stress pathways such as the UPR, and metabolic pathways such as the insulin signaling pathway (Figure 1).

The studies discussed here have linked specific neurons and neuroendocrine signals to the control of immune responses in *C. elegans*. However, they do not provide insights into the effects of pathogen infection on neuron activity or the flow of information required for the control of organismal homeostasis during immune activation. A unique advantage to neural-related studies in *C. elegans* is the unparalleled characterization of its nervous system at the cellular level. The precise and characteristic identity of each of the 302 neurons in the *C. elegans* nervous system, detailed anatomical reconstructions by electron microscopy, and functional data provide a map of the synaptic connectivity for the entire nervous system. This information will be invaluable to fully dissect the neural-immune pathways involved in the control of immune homeostasis in *C. elegans*. A detailed understanding at the molecular level of how the nervous system and the immune system cross communicate should yield potential new targets to treat not only a variety of infectious diseases but also a range of conditions that arise as a consequence of malfunctioning immune responses.
